# Growth Rate Determination of *Listeria monocytogenes* in Ready-To-Eat Fish Products Under Different Storage Conditions for Possible Shelf-Life Extension

**DOI:** 10.3390/foods14050777

**Published:** 2025-02-25

**Authors:** Paolo Cipriani, Elena Dalzini, Elena Cosciani-Cunico, Muhammad-Ehtesham Abdul, Paola Monastero, Daniela Merigo, Stefania Ducoli, Alessandro Norton, Marina-Nadia Losio, Enrico Pavoni

**Affiliations:** Istituto Zooprofilattico Sperimentale della Lombardia e dell’Emilia Romagna “Bruno Ubertini”, 25124 Brescia, Italyelena.coscianicunico@izsler.it (E.C.-C.);

**Keywords:** food safety, predictive microbiology, ready-to-eat fish products, shelf life extension, food business operators, *Listeria monocytogenes*, food pathogens, Baranyi and Roberts model

## Abstract

An increasing trend among food business operators (FBOs) to extend the shelf life of Ready-To-Eat (RTE) fish products over 5 days, the duration usually assigned to this kind of product, has been observed recently. In this study, three independent challenge tests (food artificial contamination) were performed on tuna fillet, marinated salmon tartare, and cubed salmon, with the aim of calculating the maximum growth rate (V_max_) of *Listeria monocytogenes* and estimating the time required to reach the legal limit of 2 log CFU/g, as established by European Regulation 2073/2005. The pathogen counts were fitted by the model of Baranyi and Roberts to calculate the V_max_, which were 0.041, 0.020, and 0.039 log CFU/g·h^−1^, respectively, for the tuna fillet, marinated salmon tartare, and cubed salmon at 10 °C. These results can help FBOs in assigning the correct shelf life based on hygienic practices during the process, product characteristics, and storage conditions. The time to reach the legal limit greatly depends on the starting concentration of the pathogen and on the storage temperature. The challenges for FBOs and the health authorities include reducing the contamination of *L. monocytogenes*, controlling the retail temperatures, and implementing the analytical tests for quick responses.

## 1. Introduction

Ready-To-Eat (RTE) fish products with an extended shelf life exceeding five days are increasingly available on the market, reflecting a growing trend among food manufacturers to prolong product durability. This practice necessitates an evaluation of the hygienic quality of the raw materials to ensure compliance with food safety standards. However, this has proven to be a considerable challenge because these foods are highly perishable. Among the pathogens responsible for foodborne diseases that are present in RTE fish products, *Listeria monocytogenes* is one of those of the greatest interest, given the ubiquitous distribution in the environment and in the RTE products during processing and distribution [1].

Between 1996 and 2020, *L. monocytogenes* was the second most common hazard (6.4%) among all seafood-related Rapid Alert System for Food and Feed (RASFF) notifications in Europe [2]. According to the previous studies quantifying *L. monocytogenes* in RTE fish products, the contamination levels in salmon are typically below 1 log CFU/g, although higher levels (i.e., 5–6 log CFU/g) have occasionally been detected in cold-smoked fish products [3,4]. From 2002 to 2020, seafood contamination by *L. monocytogenes* was responsible for 30% of the overall recalls and recall classifications in the United States (U.S.), with salmon and tuna accounting for 53% and 10%, respectively [5]. Additionally, *L. monocytogenes* was the primary cause for food product recalls that contained tuna [6].

According to Regulation 2073/2005, Annex I, the European Commission (EC) [7] adopts a risk-based approach, categorizing foods based on their ability to support the growth of *L. monocytogenes*, as follows:

○ Products with a pH below 4.4 or a_w_ below 0.92, as well as those with a pH < 5.0 and an a_w_ ≤ 0.94, are classified as unable to support the pathogen’s growth. Additionally, products with a shelf life of fewer than five days are included in this category. For these products, food business operators (FBOs) must guarantee that the limit of 2 log CFU/g for *L. monocytogenes* will be not exceeded at the end of the shelf life;
○ For products that can support the growth of *L. monocytogenes,* the following must be observed:
  - FBOs must prove the pathogen’s absence in 25 g of the product before the food has left their control. This criterion applies only when they are not able to demonstrate that the product will not exceed the limit of 2 log CFU/g throughout the shelf life.
  - FBOs must respect the limit of 2 log CFU/g for products placed on the market during their shelf life. This criterion applies only when the manufacturer is able to demonstrate that the product will not exceed the limit of 2 log CFU/g throughout the shelf life [7].

Otherwise, starting from 1 July 2026, for products that can support this pathogen’s growth, Reg. 2895/2024 [8] amending Reg. 2073/2005 as regards *L. monocytogenes* will request the adherence of the 2 log CFU/g limit, if the FBOs can demonstrate that the level of *L. monocytogenes* will not exceed this limit throughout the shelf life, or the absence of this pathogen in 25 g of product, if the FBOs cannot demonstrate that the level of the pathogen will not exceed the limit of 2 log CFU/g throughout the shelf life. Furthermore, FBOs may fix intermediate limits during the process that must be low enough to guarantee that the limit of 2 log CFU/g will be not exceeded at the end of the shelf life.

Due to its chemical and physical characteristics, RTE fish is a substrate that can support the growth of *L. monocytogenes*. However, regarding the shelf life, it is also necessary to consider temperature as an important factor for pathogen growth. For this purpose, the European Union Reference Laboratory for *L. monocytogenes* (EURL *Lm*), Paris, France, launched an inquiry into its National Reference Laboratory network and reviewed the scientific literature from 2002 to 2020. The outcomes were integrated into the EURL *Lm* Technical Guidance Document to assess the shelf life of refrigerated RTE foods, which resulted in the recommendation to use 10 °C as the reference temperature to simulate the reasonably foreseen storage conditions in domestic refrigerators [9]. The cold chain for RTE fish begins at the manufacturer level, where the product should ideally be stored at 4 °C or lower during the chilled storage at processing facilities [10]. However, compliance with this temperature is not always consistent. Based on the detailed data collected from FBOs, the 95th percentile of temperature observations at both the manufacturer and the retail levels is 7 °C, reflecting deviations from the recommended storage conditions.

Usually, challenge tests (artificial contamination of food with specific and selected microorganisms under controlled conditions) are performed to study the behavior of pathogens like *L. monocytogenes* in food, following the technical guidelines published by EURL *Lm* [11]. Additionally, predictive microbiology—the quantitative microbial ecology of foods—has become a crucial component of contemporary food microbiology [12].

The EC and the Codex Alimentarius [13] endorse the use of predictive microbiology in order to ensure food safety by predicting the dynamics of pathogens with reference to growth, survival, or death. Predicting the behavior of microorganisms in foods is a challenge carried out by many microbiologists to guarantee the consumers’ health.

In this paper, three challenge tests were carried out on RTE fish products—tuna fillet, marinated salmon tartare, and cubed salmon—according to the EURL *Lm* guidelines [11], and ISO 20976-1 [14] was used to calculate the maximum growth rates of *L. monocytogenes* and predict the growth in different simulated storage scenarios.

## 2. Materials and Methods

### 2.1. Inoculum Preparation

According to the recommendations of the EURL *Lm* guidelines [11], the strain 12MOB099LM (Genoserotype II, previously isolated from seafood) was selected from the EURL *Lm* set of strains characterized by their maximum growth.

The strain, stored at −80 °C in Brain Heart Infusion (BHI) broth (Oxoid Italia, Milan, Italy) supplemented with 20% glycerol, was sub-cultured in BHI at 37 °C for 24 h. A second sub-culture was prepared in BHI and incubated at 10 °C for 3 days to adapt the cell to grow in chilled conditions. The pathogen concentration was determined using ISO 11290-2 [15] to verify the concentration of this second sub-culture inoculum. Before use, the appropriate dilutions in sterile physiological solutions were prepared to obtain the inoculum at the target concentration of approximately 2 log CFU/g.

### 2.2. RTE Fish Products

The following three different RTE products were provided as single-packed units by local producers:

○ Tuna fillet: Fillet of yellowfin tuna (*Thunnus albacares*) packed in a protective atmosphere (30% O_2_–40% CO_2_–30% N_2_). Weight: Approximately 1 kg. Shelf life: 9 days at 0–4 °C.
○ Marinated salmon tartare: Chopped salmon (*Salmo salar*) marinated with sugar (0.1%) and salt (0.086%) and packed in a protective atmosphere (60% CO_2_–40% N_2_). Weight: Approximately 100 g. Shelf life: 7 days at 0–4 °C.
○ Cubed salmon: Salmon (*Salmo salar*) cubes of 1 cm^2^ packed in air atmosphere. Weight: Approximately 200 g. Shelf life: 5 days at 0–2 °C.

The products were delivered to the laboratory on the same day of production/packaging, stored at 2 ± 1 °C, and artificially inoculated within 24 h. Three different batches of each product were provided by the FBO for three weeks in a row.

### 2.3. Units Preparation

According to the EURL *Lm* guidelines [11] for each product, the following three different sample units were prepared: test units (single product packs inoculated with the subculture of *L. monocytogenes* < 1% *v*/*w*), control units (single product packs inoculated with sterile physiological solution < 1% *v*/*w*), and food control units (single product packs without inoculation). To maintain the original gas conditions in the units, the inoculation was carried out by spraying the inoculum on the surface of the products using a sterile graduated syringe and penetrating the packaging through a septum. After the inoculation, a second septum was used to cover the first one. Then, all the units were stored at 10 °C for 9 days or 7 days, respectively, for the tuna and the cubed salmon or the salmon tartare. The time of the experiments was longer then the assigned shelf life, allowing us to build the growth curves, as suggested by the EURL *Lm* guidelines. As a positive control, broth medium (BHI) was contaminated at the same concentration and incubated at 10 °C for the duration of experiments to evaluate the ability of strain to growth in these conditions. This temperature was selected as suggested by the EURL *Lm* guidelines [11] to simulate the conditions during consumer storage.

### 2.4. Microbiological and Physicochemical Analyses

Approximately 25 g of each sample (obtained by scraping the surface of the fillet tuna samples or picking up the salmon units) was transferred into a one-chamber filter stomacher bag (Neomed, Milano, Italy) and then homogenized 1:10 (*w*:*v*) in sterile peptone water (PW, Conda, Madrid, Spain) for 2 min using a Stomacher 400 blender (Seward Medical, London, UK). Decimal dilutions in sterile PW were then prepared. For the positive control in broth, 1 mL was directly diluted in PW.

For each batch, in the control and the food control units, the mesophilic lactic acid bacteria (LAB) were enumerated at the beginning and the end of storage by pour plating 1 mL of the appropriate dilution in de Man, Rogosa, and Sharpe Agar (MRSA) (Microbiol Diagnostici, Cagliari, Italy) and then incubating the plates according to ISO 15214 [16]. The pH value was measured on 10 g of sample, using an HI 223 Calibration check^TM^ Microprocessor pH meter (Hanna Instrument, Smithfield, RI, USA) equipped with a Gel-Glass electrode (Hamilton, Bonaduz AG, Switzerland). The water activity (a_w_) was measured at 25 °C with the a_w_ recorder AquaLab, series 3, Model TE (Decagon Devices, Inc., Pullman, WA, USA), following the ISO 18787 [17]. The temperature during storage was monitored and registered in one food control unit, for each batch and each product, using the Thermo Button 22 L data logger (Astori Tecnica s.n.c., Poncarale, Brescia, Italy).

For test units, 10 samples were analyzed during the storage to enumerate *L. monocytogenes* according to ISO 11290-2 [15]. Typical colonies were counted after the incubation of duplicate plates at 37 °C, respectively, for 48 or 72 h. Upon arriving at the laboratory, the detection of *L. monocytogenes* was also investigated by ISO 11290-1 [18] in a food control sample of each batch to verify the absence of natural contamination.

### 2.5. Data Analysis

The microbiological analyses of the RTE fish products and positive control were performed through a direct plate count. Microbial results were expressed as CFU/g or mL and then converted to log CFU/g or mL.

The LAB concentration, pH, and a_w_ analyses were evaluated on a single unit for three batches. Mean values were calculated as the average of three values (one for three batches). Differences in the values between the control and food control samples were evaluated at the beginning and at the end of the storage through Student’s *t*-test based on a confidence interval of 95%. The statistical analysis was performed using Microsoft Excel. The impact of the intra-batch variability of pH and a_w_ on *L. monocytogenes* growth was evaluated with the calculator tool, as suggested by ISO 20976-1 [13].

The *L. monocytogenes* concentration was evaluated on a single unit for three batches. For the test units, the fitting of the *L. monocytogenes* growth data was performed using the DMFit online version to predict and to describe the microbial growth and survival under a variety of conditions [19] to measure the growth parameters [maximum growth rate (V_max_, log CFU/g·h^−1^), Lag time (h), initial population (y_0_ log CFU/g), maximum population (y_end_, log CFU/g), coefficient of determination (R^2^), and the standard error of fit (SE of fit)] using the model of Baranyi and Roberts [20].

For each growth curve, the standard error of V_max_ was divided by V_max_ to obtain the accuracy of the estimated V_max_. If the percentage of this ratio was below 20%, the estimation could be considered accurate, as reported by EURL *Lm* guidelines [11].

In the next step, the V_max_ predicted at different temperatures (T) (predicted V_max_) was estimated according to the EURL *Lm* guidelines [11] using the simplified equation of the Ratkowsky square root secondary model [21,22] (Equation (1), as follows:

(1) $$Predicted\;V_{max}=V_{max}ref\frac{(T-T_{min}{)}^{2}}{(T_{ref}-T_{min}{)}^{2}}$$

where V_max_ref is the mean of the V_max_ estimated for each product during the challenge test at 10 °C (T_ref_), and T_min_ is the minimum growth temperature for *L. monocytogenes* (−2 °C). For each V_max_ref, the 95% confidence interval (CI) was also calculated to predict the time needed to reach the limit of 2 log CFU/g from an initial contamination of 1 log CFU/g for *L. monocytogenes*.

## 3. Results

In this work, three separate challenge tests were performed on the tuna fillet, marinated salmon tartare, and cubed salmon by contaminating the products with *L. monocytogenes* to study its growth during the shelf life of the contaminated products and to evaluate the microbiological and physicochemical changes on the uncontaminated products. The LAB concentration, pH, and a_w_ value at the beginning and the end of the storage period within the control and food control units for each RTE fish product are shown in Table 1.

High variability in the LAB concentration was observed at the beginning (t_0_) of storage between the batches, especially in the tuna fillet, where the count range was 2.1 ± 1.3 log CFU/g and 4.3 ± 2.9 log CFU/g in the control units and food control units, respectively (ranging between 1.0 log CFU/g and 6.0 log CFU/g). However, such high variability was not found at the end of the storage period (t_end_), where the concentration ranged from 6.1 ± 0.6 log CFU/g to 7.3 ± 0.7 log CFU/g. The impact of the intra-batch variability of pH and a_w_ on *L. monocytogenes* growth was not significant at the temperature condition tested, according to the ISO 20976-1 calculator [14]. The pH ranged from 5.9 ± 0.2 to 6.2 ± 0.2, with no significant differences observed among the products. Conversely, for the a_w_ (*p* < 0.05), lower values were recorded for the marinated salmon tartare, measuring between 0.95 and 0.97 with a mean value of 0.97 ± 0.01 at the beginning of the shelf life, while a value of 0.99 ± 0.01 was measured for the other products.

The storage temperature was measured and registered throughout the challenge tests at 1 h intervals, observing a mean value of 10.4 ± 0.2 °C in the tuna fillet, 10.1 ± 0.1 °C in the marinated salmon tartare, and 10.1 ± 0.1 °C in the cubed salmon.

**Figure 1:**
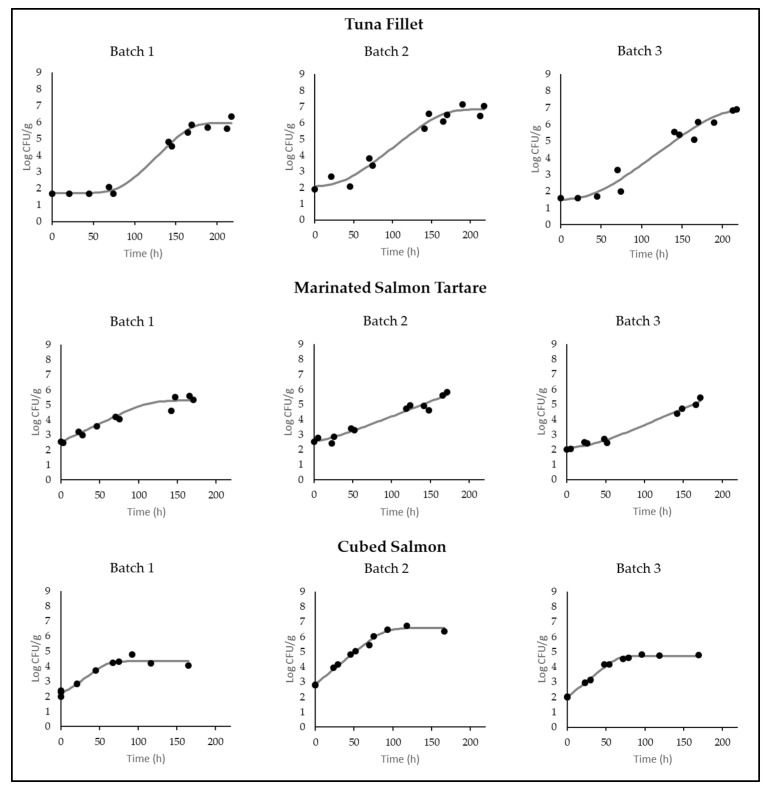
Growth curves of *L. monocytogenes* 12MOB099LM strain, fitted by the DMFit program [19] according to the model of Baranyi and Roberts [20], in three different batches for each Ready-To-Eat (RTE) fish product stored at 10 °C. Dots represent the observed data (●), and the lines represents the fit of the model. The *y*-axis reports the concentration of the strain, expressed in logarithm of colony forming unit per gram (log CFU/g), and the *x*-axis represents the time, expressed in hours (h).

After the contamination, the initial *L. monocytogenes* concentration ranged from 1.7 to 2.8 log CFU/g. The growth of *L. monocytogenes* was observed in all products, where the maximum concentration ranged from 6.9 ± 0.5 log CFU/g to 5.5 ± 0.4 log CFU/g or 6.6 ± 0.1 log CFU/g in the tuna fillet, marinated salmon tartare, and cubed salmon, respectively (Table 2). No samples showed pathogen growth at the maximum concentration of ca. 9 log CFU/g, which occurred during incubation in the positive control broth, indicating the presence of antagonists in the products, usually LAB, that compete for growth. Fitting the data with the primary model of Barany and Roberts, nine *L. monocytogenes* growth curves were obtained in three batches of tuna fillet, marinated salmon tartare, and cubed salmon stored at 10 °C (Figure 1). To get a better fit of the data, *L. monocytogenes* growth curves were obtained with the complete Baranyi model for the tuna, Baranyi model without Lag phase for the cubed salmon, and Baranyi model without Lag phase and without asymptote for the marinated salmon tartare.

Although the same *L. monocytogenes* adaptation protocol was used for all the batches in the contamination of the samples, the Lag phase was not observed for all the growth curves obtained. For example, in the tuna fillets, the Lag phase was evident only in Batch 1. The growth parameters and statistical indices of the curves are provided in Table 2.

Considering the average value of the V_max_ estimated in the three batches analyzed, the V_max_ref values obtained were 0.041, 0.020, and 0.039 log CFU/g·h^−1^, respectively, for the tuna fillets, marinated salmon tartare, and cubed salmon at 10 °C (Table 3). The uncertainty threshold was <20% for all the V_max_ estimated, and therefore they can be used to predict the growth rates at different storage temperatures.

According to the Ratkowsky model [18,19], to calculate the linear relation between the square root of the maximum growth rate and temperature (T °C), it was necessary to have at least two points—the first was the V_max_ref at 10 °C, while the second point was the V_max_ at T_min_ (−2 °C), equal to 0 for *L. monocytogenes*.

Simulating the different storage scenarios (Table 3), the square root model [18,19] was used to predict the V_max_ at different temperatures to determine the time needed to reach the critical legal limit of 2 log CFU/g, considering an initial contamination of 1 log CFU/g. The simulations were performed at 7 °C, in line with the 95th percentile of the FBO data observations [12], and at 4 °C, which was the storage temperature recommended by the manufacturers [10,23]. However, knowing the V_max_ref, other scenarios could be simulated.

## 4. Discussion

*L. monocytogenes* is an ubiquitous microorganism that may be isolated from soil, water, feces, fresh produce, food industries, and in retail environments [23,24,25]. As a consequence, many different sources can be responsible for the introduction of *L. monocytogenes* into the food chain. According to Belias et al. [26], a critical situation where preharvest contamination is relevant is the cold-smoked seafood industry, even if it is rare to identify the specific potential contamination sources of the raw material (e.g., fish farms, fish slaughter facilities, transport equipment, etc.).

Recently, the Commission Regulation (EU) 2024/2895 amending Reg. 2073/2005 as regards *L. monocytogenes* was published and is set to be transposed on 1 July 2026 [8]. To maintain consistent public health protection during the production and distribution of RTE foods, the food safety criterion “*L. monocytogenes* not detectable in 25 g” should be enforced for all instances where RTE foods are marketed throughout their shelf life. This will be applied unless the FBOs have satisfactorily demonstrated to the competent authority that the *L. monocytogenes* levels will not exceed the limit of 2 log CFU/g during their shelf life.

**Figure 2:**
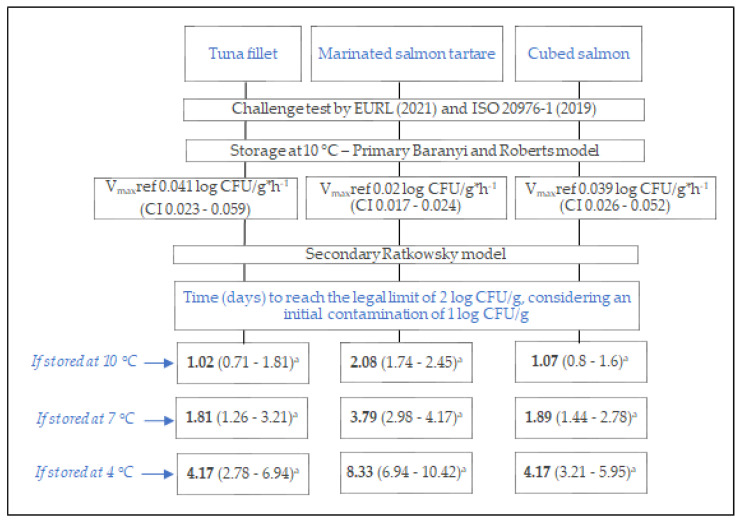
Schematic diagram illustrating the workflow, methodology, and key outcomes of this study. The V_max_ref (95% confidence interval) was calculated as the mean of the three V_max_ observed for each product during the storage at 10 °C. The Ratkowsky model was used to predict the V_max_ at other temperatures and to estimate the time (days, in bold) to reach the limit of 2 log CFU/g considering an initial contamination of 1 log CFU/g for *L. monocytogenes*. ^a^ In parentheses is the time interval, estimated by the Ratkowsky model, considering the 95% CI of the V_max_ref values observed in this study.

In this study, the growth rate of *L. monocytogenes* was estimated in RTE tuna fillets, marinated salmon tartare, and cubed salmon during storage at 10 °C. Figure 2 presents the methodology and a summary of the key results.

According to the high pH and a_w_ values (up to 5.9 and 0.97, respectively) the pathogen was able grow in these products, as reported in the literature for similar foods. Eicher et al. [4] reported the growth of *L. monocytogenes* during the storage at between 5 and 8 °C in four types of RTE salmon products with a pH of ca. 6.0 and a_w_ values ranging between 0.95 and 0.99. Wu et al. [27] observed the *L. monocytogenes* concentration in fresh tuna stored for 60 h at a static temperature of 10 °C, varying from 7.46 to 8.52 log CFU/g. Modeling the data of this study, the estimated V_max_ for each product ranged from 0.019 (in marinated salmon tartare) to 0.049 log CFU/g·h^−1^ (in tuna fillets), according to the results reported by Bolívar et al. [28], in smoked salmon pâté stored at 8 °C. It was observed that after adding sugar and salt (0.1% and 0.086%, according to the recipe) to salmon tartare, the observed a_w_ was 0.97, decreasing the growth rate by more than half of those estimated in tuna fillets and cubed salmon. This certainly increases the safety of the product but cannot be considered as a replacement for good manufacturing practices. The goodness of fit of the model was assessed by RMSE, considered as the best indicator for microbial data. The low values of RMSE (≤0.5) obtained indicate the goodness of fit, meaning that these values were close to the model predictions, as reported by Buzrul and Cam [29]. A variable concentration of LAB was observed at the beginning of the shelf life in the tested RTE fish products, with a lower level of 1 log CFU/g, until a maximum level of 5.97 log CFU/g. LAB have never been traditionally used in seafood for technological applications. Whether the presence of LAB was natural in the final product or due to contamination during processing is not yet established, and more detailed studies should be conducted to better understand and control the route of contamination [30]. Several studies in the literature have described the presence of LAB in fresh pollock, brine shrimp, gravlax fish, and vacuum-packed seafood (surimi, smoked tuna, salted cod) [31], with concentrations ranging from “not detected” to above 7 log CFU/g in cold-smoked salmon, sushi, and gravlax products [32]. Moreover, Matamoros et al. [33] screened a large selection of seafood products for the presence of LAB able to grow at 15 °C but not at 30 °C, as well as to inhibit the target pathogens. In those products, the environmental conditions allowed for the growth of LAB, as reported by Andrighetto et al. as well, who observed the presence of 3.0–4.5 log CFU/g in seafood salad, with an increasing concentration until 8 log CFU in refrigerated conditions [34]. The presence of this natural microflora can generate the so-called “Jameson effect”, i.e., the simultaneous stop in the growth of all microorganisms when the dominant population reaches the stationary phase. The hypothesis explaining this phenomenon is that the microorganisms are similarly inhibited by the production of the same by-product or by the depletion of the same nutrients. This effect was previously observed in fish and meat products [35,36], in salmon [37], fresh salmon [38], and in milk [39], describing the inhibitory effect of LAB on the growth of pathogens, mostly *L. monocytogenes*. This antagonistic effect could explain the maximum concentration level of *L. monocytogenes* observed in this study, ranging from 6.9 ± 0.5 log CFU/g to 5.5 ± 0.4 log CFU/g or 6.6 ± 0.1 log CFU/g in the tuna fillet, marinated salmon tartare, and cubed salmon, respectively, without reaching 9 log CFU/g, as obtained in the sterile broth incubated under the same conditions.

As reported by Gonzalez-Barron et al. [40], the quantitative risk assessment for RTE seafood should aim for an assessment of the control measures to reduce the frequency of cross-contamination, such as more stringent controls for raw materials, environmental monitoring programs, and/or sanitation procedures. Given the strong evidence of the inhibitory effect of the background microflora in RTE smoked fish on *L. monocytogenes*, predictive microbiology models that describe microbial competition should be employed. This study provides knowledge on the growth of *L. monocytogenes* in RTE fish products that can be used in shelf life and quantitative risk assessment studies, implementing the predictive modeling tools to establish measures aimed at controlling the growth of *L. monocytogenes* in fish products.

Recalls or other serious regulatory consequences may result from the detection of a low level of *L. monocytogenes* in a single finished product that is able to support its growth [26]. The ISO 11290-1 [18] or 11290-2 [15] are mentioned as the reference detection methods in Regulation 2073/2005, covering both the qualitative and quantitative food safety standards for *L. monocytogenes*. Because they involve a double enrichment in selective broths, these standardized methods for *L. monocytogenes* outlined in Regulation 2073/2005 are very time-consuming, taking up to 6 (–11 days) for analyses. Nonetheless, in the framework of the official controls, certain European member states have adopted the use of alternative validated test methods, such as DNA detection and amplification followed by cultural vitality confirmation. These alternative techniques take only 1 to 3 days to be performed. For this reason, the duration of *L. monocytogenes* analysis is critical for RTE products with a very short shelf life, because the microbiological safety of those already placed on the market cannot always be assured. In fact, the time needed to reach the 2 log CFU/g limit in the food matrix depends greatly on the initial concentration. However, the time spent obtaining this information by the ISO standard methods often exceeds the shelf life of the products already placed on the market.

## 5. Conclusions

In conclusion, increasing the shelf-life duration for perishable RTE fish products should be evaluated by FBOs, which must always ensure that preventive measures are respected to reduce the risk of *L. monocytogenes* contamination. Food safety will increasingly rely on predictive models of *L. monocytogenes* development. This study’s findings demonstrate the ongoing need for investigation into the microbiological safety of fish products. The presented outcomes can be useful instruments for FBOs to implement and maintain procedures to fulfil the national and international regulations. The competent authorities and FBOs have substantial stakes in reducing *L. monocytogenes* contamination, as well as in the implementation of new strategies to reduce listeriosis cases in humans (e.g., reducing the growth of *L. monocytogenes* in foods, education campaigns targeting susceptible consumers). For products with a very short shelf life, the risk is associated with the initial contamination level, and the challenge for the food industry is in reducing the contamination and proliferation of *L. monocytogenes*. Maintaining the cold chain control “from farm to fork” is considered essential to reducing the impact of possible contamination, especially for those products favoring the microbial growth due to chemical and physical intrinsic factors. Finally, the implementation of test methods that allow for reliable results to be quickly obtained, as well as the development of analytical techniques related to a change in the current legislation, will contribute to the protection of public health.

## Figures and Tables

**Table 1:** Lactic acid bacteria (LAB) concentration, expressed as logarithm of colony forming unit per gram (log CFU/g), pH, and activity water (a_w_) values measured in three batches of Ready-To-Eat (RTE) fish products at the start of the experiment (t_0_) and at the end (t_end_) at 10 °C. Values are the mean ± standard deviation of three data points (one sample for three batches). For the different times (t_0_ and t_end_), values bearing different lowercase letters are significantly different (*p* < 0.05). For the different samples (control units and food control units) at the same times, values bearing different uppercase letters are significantly different (*p* < 0.05).

RTE Fish Product	Sample	LAB (log CFU/g)		pH		a_w_	
		t_0_	t_end_	t_0_	t_end_	t_0_	t_end_
Tuna fillet	Control unit	2.1 ± 1.3 (a,A)	7.3 ± 0.7 (b,B)	5.9 ± 0.2 (a,A)	6.2 ± 0.3 (a,B)	0.99 ± 0.01 (a,A)	0.98 ± 0.01 (a,B)
	Food Control Unit	4.3 ± 2.8 (a,A)	6.1 ± 0.6 (a,B)	5.9 ± 0.1 (a,A)	5.9 ± 0.1 (a,B)	0.99 ± 0.01 (a,A)	0.98 ± 0.01 (a,B)
Marinated salmon tartare	Control unit	4.2 ± 0.1 (a,A)	6.6 ± 0.4 (b,B)	5.9 ± 0.1 (a,A)	5.9 ± 0.1 (a,B)	0.97 ± 0.01 (a,A)	0.96 ± 0.01 (a,B)
	Food Control Unit	4.2 ± 0.1 (a,A)	6.7 ± 0.3 (b,B)	5.9 ± 0.1 (a,A)	5.9 ± 0.1 (a,B)	0.97 ± 0.01 (a,A)	0.96 ± 0.01 (a,B)
Cubed salmon	Control unit	3.7 ± 0.1 (a,A)	6.3 ± 0.3 (b,B)	6.2 ± 0.1 (a,A)	6.0 ± 0.1 (a,B)	0.99 ± 0.01 (a,A)	0.99 ± 0.01 (a,B)
	Food Control Unit	3.7 ± 0.1 (a,A)	6.5 ± 0.3 (b,B)	6.2 ± 0.1 (a,A)	6.1 ± 0.3 (a,B)	0.99 ± 0.01 (a,A)	0.99 ± 0.01 (a,B)

**Table 2:** Values of hours of Lag phase (h), observed maximum growth rate (V_max_), initial concentration (y_0_), final concentration (y_end_) in log CFU/g, coefficient of determination (R^2^), standard error of fit (SE of fit) and the root mean squared error (RMSE) of the growth curves of *L. monocytogenes* at 10 °C; * not available (–).

RTE Fish Product	Batch	Lag Phase (h)	V_max_ (log CFU/g·h^−1^)	y_0_ (log CFU/g)	y_end_ (log CFU/g)	R^2^	SE of Fit	RMSE
Tuna fillet	1	82 ± 14	0.049 ± 0.009	1.7 ± 0.2	5.9 ± 0.1	0.98	0.27	0.2196
	2	37 ± 19	0.038 ± 0.006	2.1 ± 0.4	6.8 ± 0.3	0.95	0.46	0.3976
	3	41 ± 22	0.035 ± 0.006	1.5 ± 0.4	6.9 ± 0.5	0.94	0.51	0.4143
Marinated salmon tartare	1	*–	0.019 ± 0.003	2.6 ± 0.2	5.5 ± 0.4	0.94	0.27	0.2694
	2	17 ± 20	0.020 ± 0.002	2.6 ± 0.1	*–	0.95	0.27	0.2415
	3	31 ± 13	0.022 ± 0.002	2.1 ± 0.1	*–	0.98	0.18	0.1499
Cubed salmon	1	*–	0.033 ± 0.001	2.2 ± 0.1	4.3 ± 0.1	0.95	0.24	0.1951
	2	*–	0.042 ± 0.002	2.8 ± 0.1	6.6 ± 0.1	0.99	0.16	0.1366
	3	*–	0.042 ± 0.002	2.0 ± 0.1	4.7 ± 0.1	0.99	0.1	0.0871

**Table 3:** Predicted shelf life of the Ready-To-Eat (RTE) fish products, as the time to reach the legal limit of 2 log CFU/g for *L. monocytogenes,* considering an initial contamination of 1 log CFU/g. Temperature (°C), reference maximum growth rate (V_max_ref), predicted maximum growth rate (Predicted V_max_) in log CFU/g·h^−1^, and the predicted shelf life (days); * not available (–).

RTE Fish Product	Temperature (°C)	V_max_ref (log CFU/g·h^−1^)	Predicted V_max_ (log CFU/g·h^−1^)	Predicted Shelf Life (Days)
Tuna fillet	10	0.041	*–	1
	7	*–	0.023	2
	4	*–	0.010	4
Marinated salmon tartare	10	0.02	*–	2
	7	*–	0.011	4
	4	*–	0.005	8
Cubed salmon	10	0.039	*–	1
	7	*–	0.022	2
	4	*–	0.010	4

## Data Availability

The original contributions presented in this study are included in the article. Further inquiries can be directed to the corresponding author.
